# Single Walled Carbon Nanotube-Based Junction Biosensor for Detection of *Escherichia coli*

**DOI:** 10.1371/journal.pone.0105767

**Published:** 2014-09-18

**Authors:** Kara Yamada, Chong-Tai Kim, Jong-Hoon Kim, Jae-Hyun Chung, Hyeon Gyu Lee, Soojin Jun

**Affiliations:** 1 Department of Human Nutrition, Food, and Animal Sciences, University of Hawaii, Honolulu, Hawaii, United States of America; 2 Korea Food Research Institute, Seongnam-si, Republic of Korea; 3 Department of Mechanical Engineering, University of Washington, Seattle, Washington, United States of America; 4 Department of Food and Nutrition, Hanyang University, Seoul, Republic of Korea; Queen's University at Kingston, Canada

## Abstract

Foodborne pathogen detection using biomolecules and nanomaterials may lead to platforms for rapid and simple electronic biosensing. Integration of single walled carbon nanotubes (SWCNTs) and immobilized antibodies into a disposable bio-nano combinatorial junction sensor was fabricated for detection of *Escherichia coli* K-12. Gold tungsten wires (50 µm diameter) coated with polyethylenimine (PEI) and SWCNTs were aligned to form a crossbar junction, which was functionalized with streptavidin and biotinylated antibodies to allow for enhanced specificity towards targeted microbes. In this study, changes in electrical current (ΔI) after bioaffinity reactions between bacterial cells (*E. coli* K-12) and antibodies on the SWCNT surface were monitored to evaluate the sensor's performance. The averaged ΔI increased from 33.13 nA to 290.9 nA with the presence of SWCNTs in a 10^8^ CFU/mL concentration of *E. coli*, thus showing an improvement in sensing magnitude. Electrical current measurements demonstrated a linear relationship (R^2^ = 0.973) between the changes in current and concentrations of bacterial suspension in range of 10^2^–10^5^ CFU/mL. Current decreased as cell concentrations increased, due to increased bacterial resistance on the bio-nano modified surface. The detection limit of the developed sensor was 10^2^ CFU/mL with a detection time of less than 5 min with nanotubes. Therefore, the fabricated disposable junction biosensor with a functionalized SWCNT platform shows potential for high-performance biosensing and application as a detection device for foodborne pathogens.

## Introduction

The presence of pathogenic bacteria in our food and water is a major concern in the food industry because of its critical impact on public health and economy. Occurrence of foodborne illness outbreaks continues to rise as a consequence of globalized food supply, large-scale food production, and a growing population of disease susceptible consumers [1]. Each year in the United States, there is an estimated 48 million new cases of food-related illness, resulting in 128,000 hospitalizations and 3,000 deaths [2]. Consequently, annual foodborne illnesses can cost $31.2–76.1 billion for medical costs, productivity losses, and illness-related mortality [3]. Non-typhoidal *Salmonella* spp., *Listeria monocytogenes*, *Campylobacter* spp., *Escherichia coli* O157:H7, *Clostridium perfringens* and *Staphylococcus aureus* are commonly found to be the source of bacterial contaminations in our food supply [2]. Illnesses related to these pathogens range in severity from nausea and diarrhea to life-threatening conditions, such as hemorrhagic colitis and hemolytic uremic syndrome caused by *E. coli* O157:H7.

Many efforts have been made by food regulatory agencies and manufacturers to minimize the risks for foodborne illnesses, such as implementing good agricultural practices, good manufacturing practices, and hazard analysis and critical control point programs [4]. Yet, reducing the occurrence of microbial contamination remains a challenge. Therefore, detection methods play a significant role in aiding to prevent and identify foodborne pathogens.

Currently, conventional culturing techniques as well as enzyme-linked immunosorbent assay (ELISA) and nucleic acid-based polymerase chain reaction (PCR) technology are used to detect and identify pathogens. However, they are not suitable for rapid detection as they are time consuming, laborious, costly, and require stationary laboratory settings [5], [6].

Biosensing technology for food safety monitoring is a promising alternative, owing to its potential for rapid, sensitive, simple, low-cost and portable detection [7]. In particular, there is a growing interest in nano-based sensors that integrate nanomaterials into biological systems for improved sensitivity and response time. Among the nanomaterials, single walled carbon nanotubes (SWCNTs) have emerged as building blocks for nanosensor platforms [8], due to their unique mechanical, electrical, chemical, and structural properties [9], [10]. SWCNTs are hollow cylindrical tubes composed of a rolled graphite sheet. Enhanced sensing performance from the integration of SWCNTs in biosensors is attributable to its bio and size compatibility [11], structural flexibility [12], low capacitance, and axial electrical conductivity [13]. Thereby, SWCNTS can amplify the electrochemical reactivity of biomolecules [10], as it is sensitive towards minute variations in its surrounding environment [8], [11].

As a result of their unique characteristics, electrical properties of SWCNTs have been explored to study the interaction between biomolecules and nanoparticles [9], [10], [14]. SWCNT-based sensors have been fabricated based on field effect transistor (FET) designs, in which, either individual or networks of SWCNTs serve as electron channels between source and drain electrodes [11], [15]–[17]. SWCNT-FET biosensors have been applied for the detection of food pathogens because of its ability to detect changes at its interface from adsorption of charged species, [18], [19]. However, considering their intricate design and fabrication process, nano-FET biosensors face the challenge of sensor reproducibility. SWCNTs have also been integrated into electrochemical immunosensors for electrode surface modification as a means to improve electron transfer rates and working surface area [20], [21]. Studies have also used nanotubes to construct molecular junctions due to its ability to control the energy gap of electrons. Various bio and chemical sensors made of nano-junctions offer sensitivity and specificity for analytes, including glucose and heavy metal ions [22]–[25]. Despite potential applications, to our knowledge, bio-nano based junctions for detection of foodborne pathogens are not represented in literature.

This paper describes the development and performance of a disposable biosensor based on SWCNT-coated microwires assembled into a crossbar junction and immobilized with antibodies for bacterial detection. The developed biosensor operates by fabricating and optimizing a bio-nano modified surface to convert molecular binding events at the junction between target antigens and antibodies into measurable electrical signals. The key objective was intended to develop and explore the sensor's performance in detecting *E. coli* K-12 as the model pathogen.

## Materials and Methods

### Materials

7% gold-plated tungsten wire, 50 µm in diameter, was supplied by ESPI Metals (Ashland, OR). Ultem polyetherimide, mica sheets, stainless steel flat head slotted machine screws, and nuts (McMaster-Carr, Santa Fe Springs, CA) and polydimethylsiloxane (PDMS; Sylgard 184, Dow Corning, Midland, MI) were purchased for sample well fabrication. Alcohol was procured from VWR (BDH, 95%, West Chester, PA). SWCNTs with 1.5 nm diameters and 1–5 µm lengths, respectively, were purchased from NanoLab, Inc. (Waltham, MA). N,N-dimethylformamide (DMF) and polyethylenimine (PEI; 50% water solution) were supplied from Sigma Aldrich (St. Louis, MO). Streptavidin (1 mg/mL) was acquired from Thermo Scientific (Waltham, MA). Biotinylated *E. coli* antibodies (4 mg/mL) were purchased from Pierce Biotechnology, Inc. (Rockford, IL). Stock cultures of *Escherichia coli* K-12 and *Staphylococcus aureus* were obtained from the Food Microbiology Laboratory collection (University of Hawaii, Honolulu, HI).

### Instrumentation

Microwire sanitization and SWCNT dispersion in DMF was performed using a digital sonifier (450, Branson, Danbury, CT). An automated XYZ stage controlled by the COSMOS program (Franklin Mechanical & Control Inc., Gilroy, CA; Velmex, Inc., Bloomfield, NY) was used for controlling the motion of the wire during the PEI-SWCNT coating process. For the sensing measurements, a function generator (33220A, Agilent, Santa Clara, CA) and picoammeter (6485, Keithley, Cleveland, OH) was integrated into the sensing device.

### SWCNT functionalization

To create a desired SWCNT suspension, a mass of 0.1 mg of SWCNT was added per milliliter of DMF. Ultrasonic dispersion of the purified SWCNT bundles was carried out for 4 h. Microwire electrodes were cut to 33 mm and sonicated in DI water, followed by 70% alcohol. Cleaned wires were mounted onto the automated XYZ stage controlled by the COSMOS program for step-wise PEI-SWCNT surface modification. Wires were immersed into PEI solution for 5 min and withdrawn at a constant velocity of 5 mm/min. PEI coated wires were baked at 150°C in a furnace (Thermolyne, Thermo Scientific, Waltham, MA) for 10 min. Subsequently, wires were re-mounted onto the XYZ stage and immersed into the SWCNT-DMF suspension for 5 min and withdrawn at the same velocity. SWCNT dip coating was repeated once more to achieve a SWCNT network on the surface of the PEI layer. The PEI-SWCNT coating was approximately 0.84 µm thick.

### Sensor fabrication

**Figure 1:**
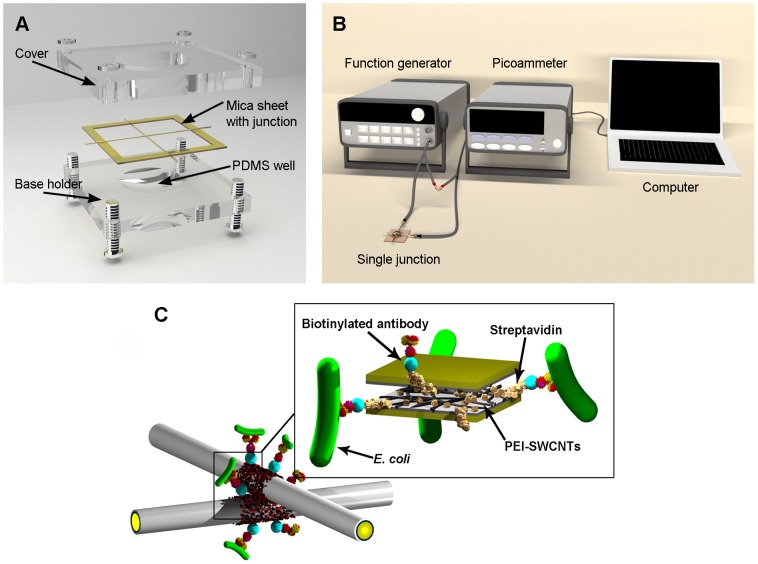
Sensor design and set-up. (A) Schematic of the single junction sensor device. Inset: FESEM image of junction. (B) Experimental configuration for electrical measurement using a junction sensor. (C) Illustration of *E. coli* captured on functionalized junction.

A bio-nano crossbar junction sensor was used in the experiments to detect *E. coli* K-12 in solution. The biosensor was fabricated with two PEI-SWCNT coated microwires assembled in an orthogonal fashion and fixed on opposite sides of a 30×30 mm mica frame, creating a 10 µm gap, respectively, between the two wires. The mica frame with attached wires was placed on an Ultem base holder (26×26×5 mm) containing a PDMS sample well and secured with an Ultem cover (Figure 1 (A)). Based on previous antibody immobilization biosensor studies [26]–[28], sensor functionalization was achieved by applying 5 µL of streptavidin to the wires' junction for 5 min followed by 5 µL of biotinylated anti-*E. coli* antibodies for another 5 min. The functionalized junction sensor was washed with sterile deionized (DI) water to remove excess antibody solution. After each experiment, the bio-nano junction sensor was autoclaved and safely discarded. A new sensor was fabricated for each trial.

### Bacterial cultivation

All experiments were performed in a certified Biological Safety Level II laboratory. 100 µL of each isolate was cultured separately in 10 mL of tryptic soy broth (TSB; BD diagnostic systems, Franklin Lakes, NJ) and incubated for 24 h at 37°C. The initial concentrations of *E. coli* K-12 and *S. aureus* stock cultures were obtained by serial dilutions and plate counting methods.

### Signal measurement

For signal measurement, the prepared biosensor was connected to a function generator for voltage input and a picoammeter for electrical current readings (Figure 1 (B)). Before reaction, 10 µL of DI water was placed on the functionalized junction, and the electric current (I_antibody_) was measured to determine negative control readings. Then a 10 µL aliquot of serial diluted *E. coli* was added to the junction sensor for 1 min to allow antibody-antigen reactions to occur (Figure 1 (C)). After the reaction, samples were washed with DI water to reduce non-specific binding effects, and the current of the sample (I_antibody-antigen_) was measured in 10 µL of DI water. DI water was used as the medium instead of a conventional electrochemical buffer to eliminate the need for a reference electrode [29]. Though electrochemical buffers are common media for electric measurements, it is typically used in three-electrode systems because electrochemical reactions are well characterized when a reference electrode is present. However, precise control of the distance between the bio-nano modified microelectrodes and reference electrode would be a challenge. In addition, ionic concentration of a buffer solution can vary depending on temperature and humidity, thus requires calibration by a reference electrode.

### Sensitivity and specificity testing

Sensitivity testing was conducted to determine the biosensor's limit of detection. A series of 10-fold serial dilutions was prepared from the stock culture using 0.1% peptone water (BD diagnostic systems, Franklin Lakes, NJ). Serial dilutions of *E. coli* K-12 were tested with the junction sensor functionalized with *E. coli* antibodies. Specificity testing was performed to determine the ability of the sensor to select for target pathogens. *S. aureus* dilutions (10^4^ CFU/mL) were tested using the *E. coli* functionalized sensor.

### Data analysis

Three replications were performed for each experiment (n = 3). To determine the sensor's ability to transduce the biorecognition reactions between antibody and antigen into measurable signals, the change in electric current (ΔI) was recorded. The ΔI signal response was determined by calculating the difference between the electrical current output of the negative control (I_antibody_) and that of the sample (I_antibody-bacteria_), as given by
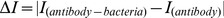
(1.1)Standard deviations of the current changes were expressed as error bars in the corresponding graphs.

### FESEM imaging

A field emission scanning electron microscope (FESEM) (S-4800, Hitachi High Technologies America, Inc.) was used to visualize the sensor's surface, before and after the capture of *E. coli* cells. Before loading the samples into the FESEM, each sample was attached to a conductive carbon tape adhered to an aluminum stub and pretreated in a Hummer 6.2 sputter coater for 40 s to achieve a thin layer of gold/palladium.

## Results and Discussion

### FESEM observations

**Figure 2:**
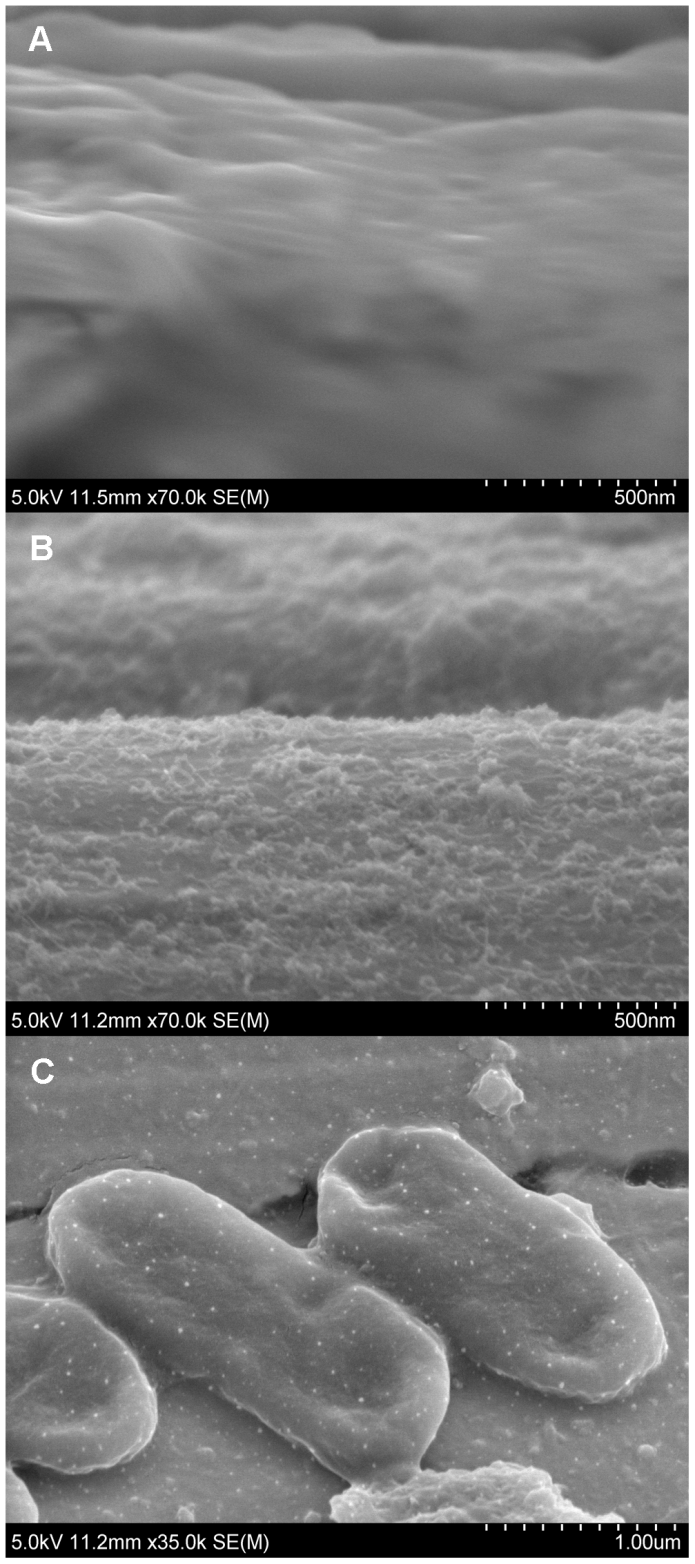
FESEM images of the sensor's microwire electrodes. (A) Bare microwire surface in comparison to (B) a surface coated with a PEI-SWCNT composite layer at 500 nm. (C) Modified surface with *E. coli* cells bound to immobilized antibodies at 1 µm.

FESEM was used to observe the surface properties of the microwire and verify the binding of *E. coli* to immobilized antibodies. Figure 2 (A) shows the surface of a bare microwire at a 500 nm scale. Modification of the bare surface with PEI and SWCNTs is demonstrated in Figure 2 (B). SWCNT colloids were deposited onto the surface of the PEI layer to form a nano-coating. The amine groups in PEI possess a high binding affinity for SWCNTs, which creates high amine nanotube interactions [30]. Due to the cationic nature of PEI and hydrophobic nature of SWCNTs, negatively charged streptavidin readily adsorbs onto the PEI-SWCNT modified surface via electrostatic and hydrophobic interactions [28], [31]. Then, biotinylated antibodies non-covalently bind to streptavidin, as a result of streptavidin's tetravalency for biotin [18], [32]. Sensor functionalization with streptavidin and biotinylated anti-*E. coli* antibodies was validated as *E. coli* cells successfully immunoreacted on the biocompatible surface (Figure 2 (C)).

### I-V curve

**Figure 3:**
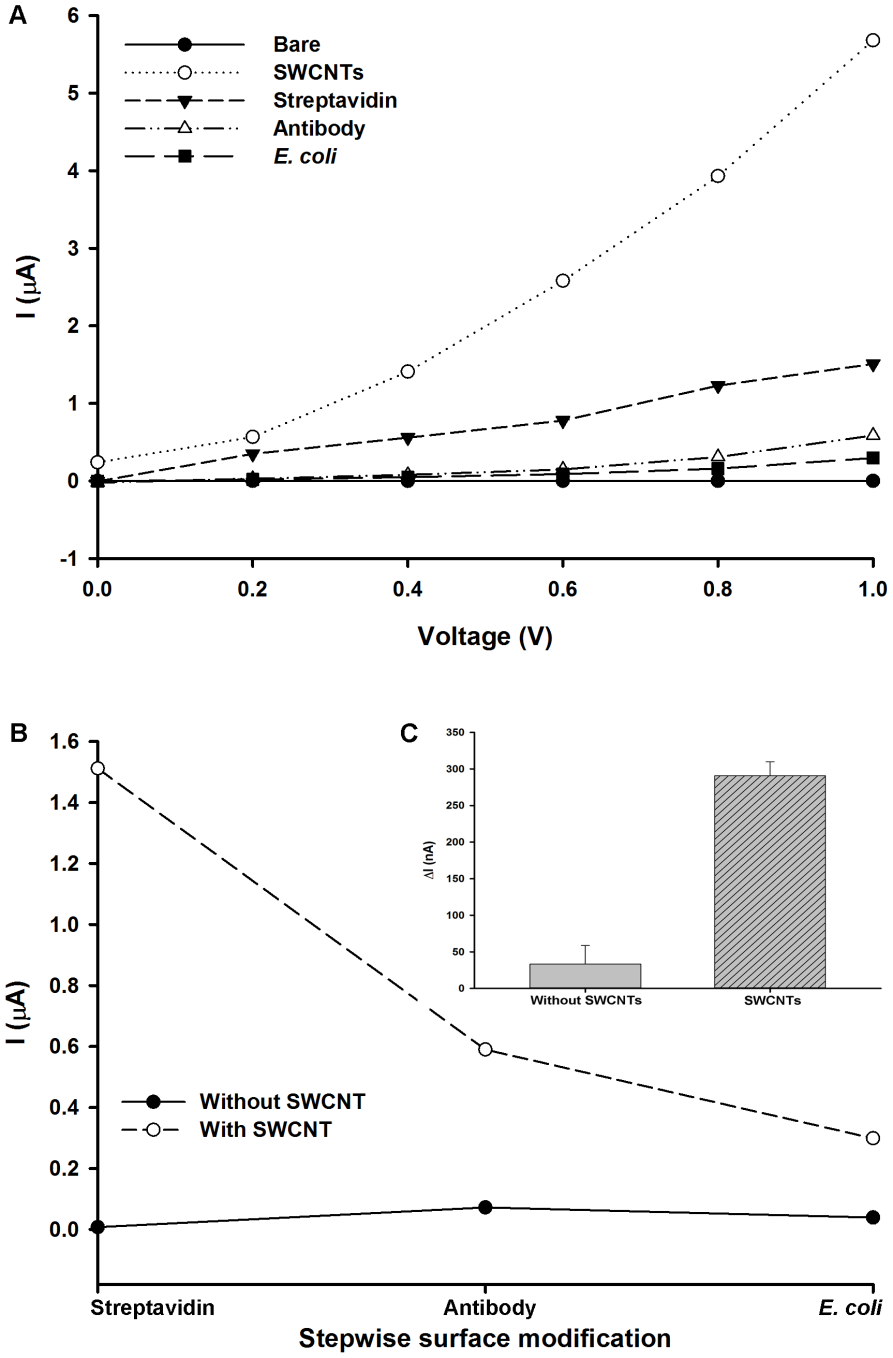
Electrical current response to step-wise modification of junction sensor. (A) I-V curve from 0 to 1 V_DC_ corresponding to individual sensor modification layers. (B) Effect of SWCNTs on signal response during functionalization and detection at 1 V_DC_. (C) Averaged change in current in response to captured *E. coli* for sensors with and without SWCNTs. *E. coli* concentration was 10^8^ CFU/mL.

Surface morphology of the junction sensor is an important factor affecting the signal response. Figure 3 (A) shows the I-V responses for the bio-nano junction sensor throughout the step-wise functionalization and detection process. An input voltage was swept from 0 to 1 V_DC_ to study the electrical response of the functionalized layers [29]. The electrical measurement was conducted individually for each layer. The average current at 1 V_DC_ for a bare, non-coated junction was 0.001 µA. Followed by an increase to 5.68 µA after SWCNTs were introduced to the surface of the sensor. The dramatic increase in current demonstrated that the SWCNT layer functioned as a conductor for electron transfer [8]. Once the junction was functionalized with streptavidin, the current decreased to 1.51 µA. Current further reduced to 0.59 µA when the biotinylated antibodies were applied to the streptavidin layer, and 0.29 µA after exposure to *E. coli* (10^8^ CFU/mL). Since the electronic properties of SWCNTs are a strong function of its atomic structure, surface modification may have induced changes in the electrical conductance of the nanotubes [13]. Protein adsorption on SWCNTs have been observed to cause a drop in conductance due to electrostatic disturbances from the biomolecules as they modulate the work-function and band alignment of the SWCNT network [11], [16], [33], [34]. Therefore, the sensor's ΔI measurements may be dependent on the surface modification of the junction surface.

The effect of SWCNTs on the sensing magnitude was also evaluated. Figure 3 (B) demonstrates a greater suppression of current at 1 V_DC_ during the functionalization process and bacterial detection when SWCNTs were integrated into the sensor. Thus, a larger ΔI of 290.9 nA was measured using a SWCNT-modified junction sensor with a 10^8^ CFU/mL *E. coli* solution (Figure 3 (C)). Whereas, a ΔI of 33.13 nA was measured from a junction sensor without SWCNTs and applied to the same *E. coli* concentration. The results indicated that the network of SWCNTs enhanced the signal response by seven-folds. The greater ΔI may be attributed to the change in electronic structure of SWCNTs occurring as a result of functionalization and *E. coli* cell loading. In addition, the SWCNTs may have enlarged the surface area of sensor, thereby imparting a higher sensitivity towards *E. coli* [20].

### Sensitivity

**Figure 4:**
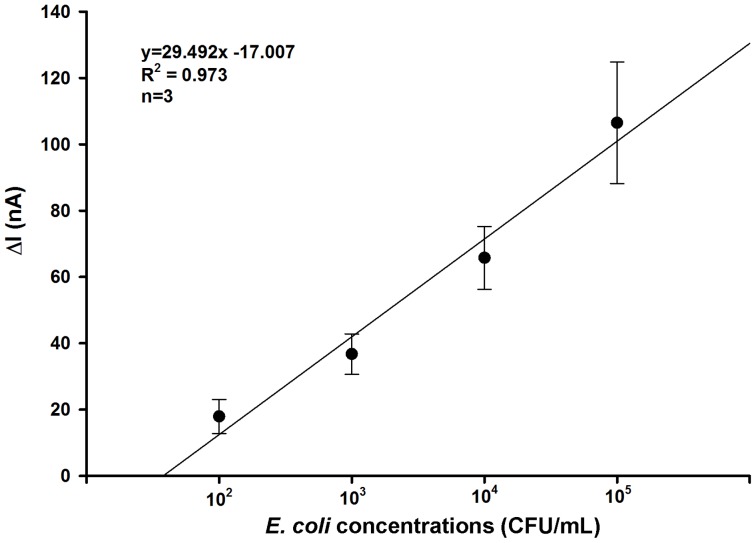
Linear relationship between changes in current and concentrations of *E. coli* (10^2^–10^5^ CFU/mL) bound to the functionalized junction sensor.

For the sensitivity test with the concentrations of *E. coli* K-12 (10^2^–10^5^ CFU/mL), electrical current measurements demonstrated a linear relationship (R^2^ = 0.973) between the change in current (ΔI) and concentrations of *E. coli* suspension in the range of 10^2^–10^5^ CFU/mL (Figure 4). Current values decreased as cell concentrations increased, hence, an increase in ΔI in relationship to the increased bacterial loadings on the bio-nano modified surface. The cause for this finding is not yet fully understood, but the phenomenon in which binding sites of antibodies on the junction become saturated with antigen concentrations thereby inducing a greater degree of change in the SWCNT electrical properties, may be partly responsible. The detection limit of the developed sensor was 10^2^ CFU/mL with a detection time of less than 5 min.

### Specificity

**Figure 5:**
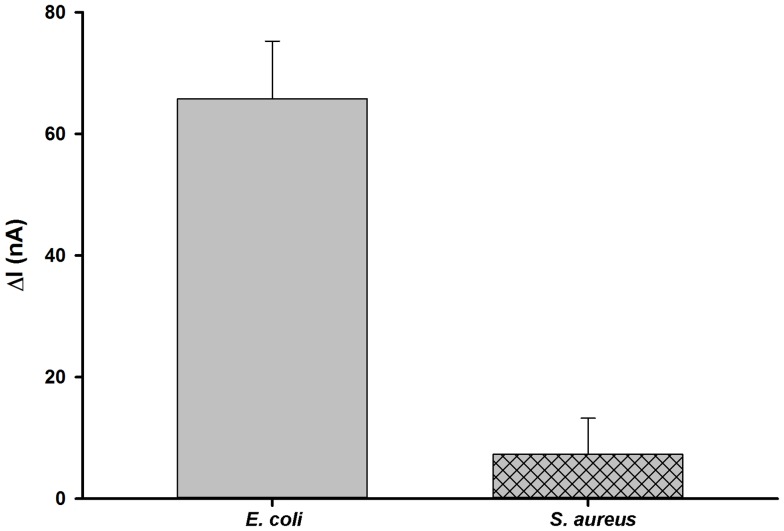
Relative response of the junction sensor to *E. coli* (10^4^ CFU/mL) and *S. aureus* (10^4^ CFU/mL).

Specificity of the sensor towards *E. coli* was measured against a pure culture of *S. aureus* (10^4^ CFU/mL). After *S. aureus* was applied to the sensor, the value of ΔI was calculated as 7.28 nA, which may be attributed to non-specific binding of *S. aureus* on the junction surface (Figure 5). However, a larger ΔI of 65.76 nA was measured with *E. coli* (10^4^ CFU/mL), indicating the specificity of the functionalized sensor towards *E. coli*.

## Conclusions

In conclusion, a functionalized SWCNT-based junction sensor was presented for label-free, rapid, and sensitive detection of *E. coli* K-12. Through experimental evidence, incorporation of SWCNTs into the sensing platform improved the sensor's signal response. The detection methodology relied on the unique properties of SWCNTs as SWCNTs could increase the sensing surface area for molecular recognition while serving as a sensing element, sensitive to surface modification. Sensitivity and specificity of the sensor was determined by monitoring the changes in electric current after bacterial cells attached to the junction surface. A linear trend of increasing ΔI was observed when *E. coli* concentrations increased logarithmically from 10^2^-10^5^ CFU/mL. In addition, little changes of ΔI were measured when the sensor was tested with *S. aureus*, which validated the sensing specificity towards *E. coli* cells. Therefore, the junction sensor offers advantages over conventional detection assays, in that the sensor can be readily fabricated to exhibit sensitivity and specificity with a 5 min detection time. We expect that the developed sensor will provide a promising approach for pathogenic detection in serially diluted cultures, and furthermore can be integrated into a portable multiplexed device used not only in food safety industries, but in agricultural and bioterrorism industries as well.
